# Recognizing the importance of new tools and resources for research

**DOI:** 10.7554/eLife.07083

**Published:** 2015-03-31

**Authors:** Randy Schekman, Detlef Weigel, Fiona M Watt

**Keywords:** scientific publishing, peer review, experimental tool and technique, data and other resource, eLife

## Abstract

*eLife* is introducing a new article type—called Tools and Resources—to highlight new experimental techniques, datasets, software tools and other resources.

The scope of a journal has many dimensions. The obvious one is scientific: does the journal accept submissions in a particular area? In this regard *eLife* aspires to encompass all of the life sciences. Another more contentious dimension is quality: is a given paper significant enough for the journal in question? A recent *eLife* editorial addressed this matter (Malhotra and Marder, 2015): ‘For us,’ the article explained, ‘the ideal *eLife* paper presents an accurate description of data that makes others in the field think differently and moves the field forward’. Crucially, there are no constraints on the number of papers that can be published in *eLife*: we accept all the papers that meet our standards (Schekman et al., 2013). A third dimension concerns the types of article that a journal publishes. The National Library of Medicine in the US, for example, recognizes a remarkable 30 different types of article, from abstracts and addendums to review articles and translations. PubMed offers even more ‘publication types’. It is this aspect of scope that we discuss here.

When *eLife* opened for submissions in the middle of 2012 we offered just one article type for peer-reviewed, original research. Since then the *Research Article* has been joined by several other formats. *Short Reports*, as their name suggests, are short pieces of original research: although there is no minimum length for a *Research Article*, authors submitted relatively few short manuscripts in the early days of the journal, so we created a specific format to encourage shorter submissions. *Research Advances* are papers that build on previously published eLife articles in an important way (Patterson et al., 2014). The *Registered Report* has emerged as part of efforts to improve the reproducibility of research (Errington et al., 2014): in essence it is an experimental protocol that explains how its authors intend to replicate a previously published result. In due course the results of these experiments will be published in a *Replication Study*.

With this editorial, we are introducing the *Tools and Resources* format, which encourages authors to publish details about new techniques or resources that have the potential to lead to important breakthroughs in one or more areas of the life sciences. The publication of *Tools and Resources* articles reflects the increasing sophistication of the experimental equipment and approaches used to probe biological systems, and the exponential growth in the volume of data generated by new methods, reagents and technology.

The first *Tools and Resources* articles to be published provide a flavour of what is to come. Mayeul Collot and colleagues report a new high-affinity calcium probe called CaRuby-Nano for use in cell biology and neuroscience, including dual-colour imaging experiments (Collot et al., 2015), and Hugo Bellen and colleagues describe a genetic library of hundreds of GFP-tagged proteins, and their use in a variety of experiments on Drosophila (Nagarkar-Jaiiswal et al., 2015). As these examples illustrate, we welcome the submission of significant technological or methodological advances, including genomic or other datasets (such as brain atlases), collections of biological resources, software tools, and so on, especially when linked to examples that demonstrate their broad utility.

*Tools and Resources* articles should fully describe the biological material, data and methods so that prospective users have all the information needed to deploy them within their own work. Therefore, major datasets must be publicly deposited (unless there are strong ethical reasons to restrict access); biological resources must be available from stock centers; relevant program code must conform to the Open Source Definition and be deposited in an appropriate public repository; and methodological advances must be comprehensibly described, along with details of the reagents and equipment, and their sources.

*Tools and Resources* articles should fully describe the biological material, data and methods so that prospective users have all the information needed to deploy them within their own work.

The peer-review process for *Tools and Resources* is the same as that used for *Research Articles*, *Short Reports* and *Research Advances*. However, the selection criteria are different. *Tools and Resources* articles do not have to report major new biological insights or mechanisms, but it must be clear that they will enable such advances to take place. Specifically, these contributions will be assessed in terms of their potential to facilitate experiments that address problems that to date have been very challenging or even intractable.

The creative energy and painstaking work that go into the development of new research tools, and the extra mile that many researchers go to make these resources available and reusable by their communities, deserve recognition. We hope that this new article type proves to be an effective venue to celebrate these critical contributions to science.
